# Verringerung des Abfallaufkommens im Convenience-Food-Bereich – Möglichkeiten und Herausforderungen pfandbasierter Mehrwegbehälter

**DOI:** 10.1007/s00506-020-00720-6

**Published:** 2020-10-29

**Authors:** Dominik Kornthaler, Anke Bockreis

**Affiliations:** Fakultät für Technische Wissenschaften, Institut für Infrastruktur, Arbeitsbereich Umweltechnik, Abfall- und Ressourcenmanagement, Universität Innsbruck, Technikerstraße 13, 6020 Innsbruck, Österreich

**Keywords:** Kreislaufwirtschaft, Einwegverpackungen, Abfallwirtschaft, Verpackungen, Kunststoffabfälle, Pfandsystem, Nachhaltigkeit, Circular Economy, Single use packaging, Waste Management, Packaging, Plastic waste, Deposit refund scheme, Sustainability

## Abstract

Der Convenience-Food-Bereich und die entsprechenden Dienstleistungen nehmen zu. Ein Großteil der entsprechenden Produkte wird in Einwegverpackungen angeboten oder angeliefert. Dementsprechend wächst auch der Abfallberg aus diesen Einwegverpackungen weiter. Beispiele aus der Literatur und anhand von Unternehmen zur Verfügung gestellter oder öffentlich zugänglicher Informationen zeigen dies gleichermaßen auf. Nicht nur Kunststoffe, sondern auch andere Materialien tragen zur Problematik bei. Reglementierungen für Kunststoffeinwegprodukte vernachlässigen also einen großen Teil der Problematik, da auch die Kunststoffalternativen der Einwegprodukte an der zunehmenden Abfallmenge beteiligt sind. Vermeidung von Einwegprodukten stellt im Sinne der nachhaltigen Ressourcen-Nutzung, der Vermeidung des Litterings sowie der hohen Abfallentsorgungs- und Behandlungskosten die effizienteste Zukunftsstrategie dar. Deshalb werden Mehrwegbehälter für verschiedene Anwendungen im Convenience-Food-Bereich als zukunftsträchtige Alternative angesehen. Beispielsweise können pfandbasierte Mehrwegsysteme theoretisch mehr als 90 % des jährlich anfallenden Verpackungsabfalls aus dem Take-away verhindern. In der Praxis sind es jedoch unter 10 %. Zusätzlich können durch die Etablierung solcher kreislaufwirtschaftlicher Systeme Arbeitsplätze generiert werden. Die Potenziale pfandbasierter Mehrwegsysteme in Bezug auf Abfallvermeidung und Arbeitsmarkt stehen den Herausforderungen gegenüber. Diese Herausforderungen betreffen sowohl das Bewusstsein der Konsument*innen und des Verkaufspersonals, als auch systemimmanente Voraussetzungen. So steht zum Beispiel nicht überall genügend Platz für die Reinigung und Lagerung der Mehrwegbehälter zur Verfügung. Für eine erfolgreiche Nutzung pfandbasierter Mehrwegsysteme müssen also einige Voraussetzungen erfüllt werden.

Abschließend ist es das Material der Mehrwegbehälter, das ebenfalls einen großen Einfluss auf die künftige, flächendeckende Nutzung von Mehrweg- statt Einwegbehältern nehmen wird. Insbesondere die Mikroplastikthematik stellt in diesem Zusammenhang eine noch ungeahnte und wenig erforschte Größe dar.

## Abstract (en)

The market for convenience food and services, like food-delivery, takeaway, and pre-prepared meals is steadily growing. Many of these products are offered in single use packaging. Consequently, the waste produced by convenience food consumption is also growing. Information from literature and available data from companies provide are used to provide an overview of this waste. Even though plastics contribute a significant part to this waste, it is just a part of it. Additionally, the substitutes used instead of plastics nowadays are mostly designed for single-use application. In terms of resource sustainability, the avoidance of waste production should be imperative. With this respect re-use containers gain a growing interest and are discussed as a good future option for some convenience food sectors. Examples reveal that the annual waste production linked to takeaway food could be reduced by over 90% by implementation of re-use containers. Furthermore, they also reduce the cost of waste management, and create new job opportunities.

However, besides opportunities there are challenges. The theoretical waste reduction potential is high, the one gained in practice, however, is rather low. This is due to customers and sales stuff alike. Furthermore, the infrastructure required for such systems to work smoothly is not available everywhere. Especially space for cleaning, drying and storage of the re-use containers is sparse, depending on the type of facility.

Finally, questions concerning the material used for re-use containers remain unanswered yet. Especially the growing concerns about Plastic Micro- and Nanobeads remain to be a black box.

## Einleitung

Aufgrund der zunehmenden Nutzung von Convenience-Food-Produkten und -Dienstleistungen nehmen auch die entsprechenden Verpackungsabfälle zu (Istel and Detloff 2018; Kauertz et al. 2019; Schüler 2018). Neben Fast Food fallen auch verzehrfertig zubereitete oder noch in der Mikrowelle zu wärmende Speisen, Take-away und geliefertes Essen in diese Kategorie. Die aktuelle Sars-Cov-2-Krise hat diese Entwicklung verschärft und zu einer weiteren Zunahme geführt (Fischer 2020; Sokola 2020).

Die zugrundeliegenden Einflussfaktoren sind Entwicklungen in Lebens‑, Arbeits- und Denkweisen unserer Gesellschaft (Grundberg et al. 2018). Insbesondere Veränderungen am Arbeitsmarkt, wie höhere Flexibilität in Bezug auf Arbeitszeiten und die Entfernung zum Wohnort sowie die Zunahme von Singlehaushalten gehen mit der Steigerung der Convenience-Food-Nutzung einher (Schüler 2018).

Unternehmen und Netzwerke wie die PREVENT Abfall Allianz (Schinkel and Wilts 2020) oder auch die „Reloop Platform“ zeigen ein Umdenken auf, das zur Veränderung dieser „Wegwerfgesellschaft“ führen kann. Diese Veränderung ist jedoch noch mit vielen Unbekannten behaftet. Darüber hinaus entwickelt sich auch das Bewusstsein der Konsument*innen in Richtung ökologischer Einwegalternativen (Gill et al. 2020) und Mehrwegsysteme (DUH 2020).

Die Zunahme der Verpackungsabfälle stellt das Abfallmanagement von kommunaler Seite bis hin zur Gesetzgebung vor Herausforderungen. Insbesondere Kunststoffabfälle stellen, aufgrund jener Eigenschaften, die Kunststoffe zu weitverbreiteten Werkstoffen machen, eine globale Herausforderung dar. Sie sind leicht, widerstandsfähig und langlebig. Sie verbreiten sich global, trotzen Umwelteinflüssen und tun dies für Jahrhunderte (Geyer et al. 2017). Selbst wenn sie mit freiem Auge nicht mehr sichtbar sind, stellen sie aufgrund ihrer Mikrostruktur ein noch ungeahntes Gefährdungspotenzial für Mensch und Umwelt dar (Kedzierski et al. 2018).

Aktuelle umweltpolitische Maßnahmen sind dementsprechend zu begrüßen, wie z. B. das Verbot von Einwegsackerln und die EU-Richtlinie bezüglich der Limitierung leicht zu litternder Kunststoffeinwegprodukte (Christoforou 2019).

Darüber hinaus hat die EU bereits vor Jahren einen Maßnahmenkatalog erarbeitet, der die kreislaufwirtschaftliche Nutzung von Kunststoffen erleichtern soll (EC 2015). Dies limitiert auch den Kunststoffeintrag in die Umwelt. In Österreich wurde diesbezüglich vereinbart, dass der Anteil an Kunststoff-Recycling bis 2025 auf 50 % gehoben werden soll (Enzinger 2019).

Neue Technologien im Bereich des Kunststoffrecyclings werden immer häufiger als Lösung für die wachsenden Abfallberge betrachtet. Technische und systembedingte Limitierungen erschweren jedoch den Recyclingprozess (Hahladakis and Iacovidou 2018). Die stetige Weiterentwicklung chemischer Recyclingmethoden birgt zwar das Potenzial auch stark verschmutzte Kunststoffe verarbeiten zu können, ist aber noch nicht tauglich für den großtechnischen Einsatz (Lechleitner et al. 2019). Aufgrund der hohen Recyclingkosten, die sich auch auf den Produktpreis auswirken, kann Recycling theoretisch einen positiven Effekt auf die Nutzung von Einwegprodukten erzielen (Van Eygen and Fellner 2019).

Reglementierungen und technische Innovationen sind wichtige Schritte Richtung Kreislaufwirtschaft. Neben Bewusstseinsbildung tragen beide zur Reduktion eines beachtlichen Kunststoffeintrags in die Umwelt bei.

Laut Frau Christine Hochholdinger vom Bundesministerium für Nachhaltigkeit und Tourismus wurde während der ÖWAV-Tagung an der BOKU am 17.09.2019 in diesem Kontext jedoch festgehalten, dass „… aktuelle Reglementierungen Produkte, die kein Litteringpotenzial haben, ausnehmen. … dies betrifft Kunststoffverpackungen verzehrfertiger Speisen, die noch gewärmt werden müssen und Kunststoffalternativen.“

Die Substitute, die Kunststoffe ersetzen sollen, werden das Problem der Abfallmengen lediglich in eine andere Richtung verschieben. So lässt sich aus abfallwirtschaftlicher Sicht festhalten, dass bereits jetzt der größte Anteil des mit Außer-Haus-Konsum zusammenhängenden Abfalls die Fraktion Papier-Pappe-Karton (PPK) darstellt (Istel and Detloff 2018).

Eine nähere Betrachtung des deutschen Außer-Haus-Konsums ergab ein beeindruckendes Wachstum der assoziierten Dienstleistungssektoren (Istel and Detloff 2018; Schüler 2018). Dazu kommt, dass Third-Party-Delivery-Unternehmen das Angebot zu liefernder Speisen enorm vergrößert und damit das Interesse an Lieferungen erhöht haben (Grundberg et al. 2018). Third-Party-Delivery-Unternehmen sind Unternehmen, die für Restaurants ohne eigene Auslieferinfrastruktur, Logistik und Lieferant*innen anbieten, um am stetig steigenden Markt für Essenslieferungen teilhaben zu können (Grundberg et al. 2018). Wie auch Lieferunternehmen nutzen auf Take-away spezialisierte Anbieter exklusiv leichte Transportverpackungen, die derzeit meist in Form von Einwegprodukten vorliegen. Damit tragen diese Unternehmen bereits maßgeblich zu den Abfallmengen durch Einwegverpackungen bei (Schüler 2018).

Interessanterweise spielen auch Betriebsrestaurants eine immer größere Rolle in diesem Zusammenhang (Chalupova 2019). Um mit Bistros, Take-away-Restaurants und verzehrfertigen Convenience-Produkten konkurrieren zu können, bieten Betriebsrestaurants die zubereiteten Speisen immer häufiger auch als Take-away an.

Fokus dieser Arbeit ist der „Convenience Food“ Sektor, d. h. Lieferservices, Take-away und verzehrfertige Produkte aus Supermärkten.

Das Interesse an diesen Dienstleistungen aus dem Blickwinkel des Abfall- und Ressourcenmanagements ergibt sich, neben der Zunahme der Abfallmenge, aufgrund mehrerer Faktoren:

- Der gesamte Sektor des Außer-Haus-Konsums erlebt seit Jahren stetiges Wachstum.
- Einzelne Subsparten, insbesondere Third-Party-Delivery-Unternehmen, wachsen mit bis zu über 100 % pro Jahr (Delivery Hero AG 2017; Grundberg et al. 2018)
- Die zugrundeliegenden sozioökonomischen Veränderungen werden sich aus derzeitiger Sicht nicht zurückentwickeln.
- Es gibt Alternativen zu den bestehenden, auf Einwegnutzung basierenden Systemen, die jedoch noch Optimierungsbedarf aufweisen.

Anstatt Recycling von Einwegprodukten als exklusive Kardinallösung zu sehen, wird immer häufiger vorgeschlagen, auf Mehrweglösungen zu setzen, um die Abfallmenge zu reduzieren (Chalupova 2019).

So gibt es im Bereich der Coffee-To-Go-Becher bereits verschiedene Mehrweglösungen (Kauertz et al. 2019). Anhand bereits bestehender Angebote werden Herausforderungen an Mehrwegsysteme ersichtlich. Diese existieren entlang der gesamten Kette von der Materialwahl und Produktion, über die zugrundeliegende Systemlogistik, die Nutzung durch Konsument*innen und Unternehmen bis hin zum Abfallmanagement.

Das theoretische Abfallvermeidungspotenzial von Mehrweglösungen beträgt dabei über 90 % des eingesetzten Materials für Einwegverpackungen. In der täglichen Nutzung werden jedoch nur wenige Einweg- durch Mehrwegverpackungen ersetzt.

Diese Arbeit beleuchtet Herausforderungen und Möglichkeiten, die mit der Verringerung von Verpackungsabfällen durch die Einführung pfandbasierter Mehrwegbehälter für Convenience Food einhergehen. Anhand bereits laufender Projekte und aktueller Daten zu Verbrauchsmengen wird einerseits dargestellt, welche Vorteile die Nutzung von Mehrwegbehältern mit sich bringt. Andererseits werden Herausforderungen thematisiert, die pfandbasierte Mehrwegsysteme betreffen.

## Methodik

Neben Literatur bezüglich des Außer-Haus-Konsums werden auch Informationen über die Abfall- und Verpackungsmengen von Tiroler Unternehmen aus dem Convenience-Food-Bereich verarbeitet. Die Unternehmensinformationen werden auf Wunsch der entsprechenden Unternehmen ausschließlich anonymisiert dargestellt.

Verschiedene Mehrwegsysteme, die in diesem Bereich Anwendung finden, werden bezüglich der genutzten Materialien und Anwendungsbereiche betrachtet. Darunter befinden sich auch negative Beispiele, deren Konzepte bzw. Geschäftsmodelle nicht funktioniert haben.

Gespräche mit Hersteller*innen von Einwegverpackungslösungen beleuchten aktuelle Hindernisse in der Nutzung von Mehrwegbehältern für einzelne Produkte aus dem Convenience-Food-Bereich.

Weiterhin wurde eine mögliche Ersparnis an Einwegverpackungsmaterial in Tonnen pro Jahr für verschiedene Wirtschaftszweige ermittelt. Anhand dieser Informationen können darüber hinaus Herausforderungen an Mehrwegsysteme dargestellt und Vorschläge für deren effiziente Einführung unterbreitet werden.

## Ergebnisse und Diskussion

### Abfallmengen durch Convenience Food und Abfallreduktion durch Mehrwegbehälter

Das Abfallaufkommen im Bereich des Außer-Haus-Verzehrs hat in Deutschland von 1994 bis 2017 um 77.400 t zugenommen (Schüler 2018). 2017 beträgt das mit diesem Bereich assoziierte Abfallaufkommen bereits 281.186 t exklusive Haushaltsverpackungen beziehungsweise 346.831 t inklusive; 119.879 t entfallen dabei auf Menüboxen und Snackboxen wie zum Beispiel Pizzakartons, Lunchboxen, Nudelboxen etc. (Schüler 2018). Haushaltsverpackungen sind Produkte mit Verpackungsfunktion, die unbefüllt gekauft werden wie z. B. Folien, Schüsseln u. Ä. Das gesamte für 2017 ermittelte Abfallaufkommen in diesem Bereich setzt sich aus 64 % Papier-Pappe-Karton, 30 % Kunststoff, 4 % Alu und 1 % Naturmaterialien zusammen (Istel and Detloff 2018). Für die Kunststoff- und Papier-Pappe-Karton-Fraktion bedeutet dies, dass 1,8 % bzw. 2,8 % des Gesamtabfallaufkommens in Deutschland (Umweltbundesamt 2019) aus diesem Wirtschaftszweig entstammt.

Dass diese Zunahme im Bereich des durch Convenience Food verursachten Abfalls von globaler Brisanz ist, zeigen unter anderem in England, den USA und China erhobene Abfallmengen: So führt Take-away zu 13.680 t Aluminiumabfall pro Jahr in Form von 1,8 Mrd. Einwegschalen in England bzw. 58.500 t expandiertes Polystyrol in Form von 7,5 Mrd. Einwegschalen in den USA (Gallego-Schmid et al. 2019).

Für chinesische Megastädte wurde ein Anstieg der durch Essenslieferungen verursachten jährlichen Abfallmengen von 1,3 Mio. t im Jahr 2015 auf 1,5 Mio. t im Jahr 2017 ermittelt (Song et al. 2018). Im Vergleich zu Deutschland dominieren Kunststoffe das entsprechende Abfallgesamtaufkommen mit 75 %. Für Österreich sind entsprechende Informationen in dieser Auflösung nicht verfügbar. Dennoch lässt sich anhand von Studien des Ökologie Institutes (Hietler and Pladerer 2020) festhalten, dass zur Einwegnutzung konzipierte Convenience-Verpackungen einen Anteil zum jährlichen Abfallaufkommen beitragen.

Für Tirol fehlen Daten, die eine genaue Zuordnung dem Außer-Haus-Konsum bzw. dem Convenience Food erlauben ebenfalls. Die Restabfallanalyse 2019 für Tirol bietet jedoch eine Übersicht über die Menge an Leichtverpackungen (LVP), die im Zeitraum 2018/19 anfielen, so wurden 23.935 t an Leichtverpackungen getrennt gesammelt. Inklusive der Fehlwürfe von 8077 t, die im Restabfall landeten kamen so 32.012 t an LVP zustande. Darüber hinaus wurde in einem Artikel der Tiroler Tageszeitung (Heubacher 2018), in einem Interview mit Vertreter*innen der Firma Höpperger Recycling, die Kunststoffabfallmenge (exklusive Gewerbeabfall) mit 24.000 t für das Jahr 2016 angegeben.

Um abzuschätzen, welcher Anteil der Convenience-Food-Bereich zum Abfallaufkommen in Tirol beiträgt, wurde bei regionalen Unternehmen die Menge an entsprechendem Verpackungsmaterial nachgefragt. Angefragt wurde bei Unternehmen aus der Gastronomie und dem Lebensmitteleinzelhandel (LEH). Bei letzterem wird generell unterschieden zwischen verzehrfertigen Produkten (Sandwiches, Salate, Finger Food Snacks etc.) und Speisen, die zuhause oder am Arbeitsplatz gewärmt, also fertig zubereitet werden müssen. 4 Betriebe, die sowohl in Tirol als auch österreichweit tätig sind, lieferten Informationen.

Die anfallenden Mengen für die zu wärmenden Speisen ergaben bei einem Betrieb, dass schwarze PP-Tassen und vierlagige Folien mit 157,7 t pro Jahr zum LVP-Aufkommen bzw. dem Kunststoffabfallaufkommen in Tirol beitragen. In Bezug zu den Daten aus der Restabfallanalyse und den Interviews aus der TT bedeutet das, dass eine einzelne Produktpalette eines Tiroler Betriebs also bereits 0,7 % der gesamten jährlichen Kunststoffabfallfraktion bzw. 0,5 % der jährlich anfallenden LVP-Fraktion verursacht.

Zusätzlich ist in Tirol ein Trend Richtung Lieferdienste zu erkennen. Für den Zeitraum 1998–2007 konnten 3 Restaurants mit Pizzalieferservice in Innsbruck im Rahmen einer Internetrecherche ermittelt werden. Seit 2008 nahm dieses Angebot stetig zu. Diese Zunahme dürfte der Einführung der Third-Party-Delivery-Plattformen geschuldet sein, die sich seit 2008 auch in Tirol etablierten. Derzeit (Stand 2020) finden sich im WWW allein in Innsbruck 24 Anbieter für die Lieferung von Pizzen. Die Lieferung erfolgt entweder über einen eigenen Lieferdienst oder einen sogenannten Third-party-Delivery-Service (z. B. Delivery Hero).

Eine initiale Befragung, um einen Überblick über die zu erwartenden Abfallmengen zu bekommen, wurde bei zwei Betrieben durchgeführt: ein auf Auslieferung spezialisierter Pizzalieferservice und ein italienisches Restaurant, das auch „zum Mitnehmen“ anbietet.

Der auf Auslieferung spezialisierte Betrieb verbraucht pro Jahr 32.000 Pizzakartons. Bei einem angenommenen Gewicht von 115 g pro Karton (Schüler 2018) ergibt dies 3,6 t PPK Abfall pro Jahr durch diesen Betrieb. Jener Betrieb, der auf Gaststättenbetrieb spezialisiert ist, bietet ebenfalls Take-away-Pizzen an und verbraucht ca. 5600 Kartons pro Jahr. Dies entspricht, unter derselben Annahme bezüglich des Gewichts, 0,6 t PPK Abfall pro Jahr (vgl. Tab. 1). Hochgerechnet auf die 24 Pizzalieferservices in Innsbruck entspricht das 51,8 t PPK Abfall pro Jahr. Da stark verschmutzte Pizzakartons in den Restabfall gehören, ist davon auszugehen, dass ein Teil des anfallenden PPK-Abfalls seinen Weg in die Restmülltonne findet.

**Tab. 1:** Übersicht über die durch zwei verschiedenen Betriebstypen verursachten Abfälle; in der Spalte rechts wird die Abfallmenge dargestellt, die durch derzeitige Mehrwegalternativen verursacht werden würde

Betriebsart	Pizzakartons pro Jahr	Material	Gewicht in g	Abfall in g/Nutzung	Abfall in t/a	Mehrweg Abfall in t/a
Lieferdienst	32.000	Karton	115	115	3,6	1,7
Gaststättenbetrieb mit Take-away	5600	Karton	115	115	0,6	0,4

Laut der oben genannten Restabfallanalyse fielen 2018/19 in Tirol 67.389 t an getrennt gesammeltem PPK-Abfall an (Technisches Büro Hauer 2020). Daraus ergeben sich knapp 100 kg PPK Abfall pro Einwohner*in in Tirol. Rechnet man Pizzakartons komplett der getrennt erfassten PPK-Fraktion zu, machen sie ca. 0,4 % des jährlich anfallenden PPK-Abfalls in Innsbruck aus. Auf ganz Tirol bezogen tragen allein die Pizzakartons in Innsbruck mit 0,1 % zum Tiroler PPK-Aufkommen bei. Immer vorausgesetzt, dass der Verschmutzungsgrad der Kartons das Entsorgen in den Papierabfall rechtfertigt.

Aktuelle Angebote für Pizza-Mehrwegtransportverpackungen (Single 2019) können die Kartonabfallmenge pro Nutzung um 55 % reduzieren, vgl. Tab. 1. Diese Mehrweglösungen basieren auf einer mehrfach anwendbaren Polypropylen (PP)-Umverpackung und einem Einlegkarton. Im Falle dieser Pizzamehrwegverpackung verbleibt der PP-Teil bei der Lieferant*in und nur der Einlegkarton bei der Kund*in, die diesen nach Konsum entsorgt.

In Tab. 2 wird eine Übersicht einiger Anbieter von Mehrwegsystemen dargestellt. Auch wenn Pizzabow kein Anbieter im eigentlichen Sinne ist, wurde es hierfür aufgenommen, um Optionen für Pizzalieferungen aufzuzeigen.

**Tab. 2:** Beispiele verschiedener Mehrweglösungen im Take-away und Lieferbereich innerhalb des deutschsprachigen Raums

Anbieter	Region	Aktiv seit	System	Behältertyp(en)	Material(ien)	System
Tiffinloop	Berlin	2015	Pfand	Schüsseln mit Deckel	Stahl	App, PC-Software
reCircle	CH	2016	Pfand	Schüsseln mit Deckel; Becher mit Deckel	PBT, PP	App, PC-Software
Migros	CH	2017	Pfand	Schüssel mir Deckel	PBT, PP	Homepage-Infos
Rebowl	GER	2019	Pfand	Schüsseln mit Deckel	PP, TPE	App, PC-Software
Pizzabow	GER, AUT	2019	Verkauf an Lieferdienste	Pizzaschachtel, klappbar	Karton, PP	Anbieterabhängig

Im Bereich Take-away zeigt ein Beispiel aus der Schweiz auf, dass bereits eine Abfallreduktion über 90 % möglich ist. Migros, eine schweizweit operierende Lebensmittel-Einzelhandelskette, bietet in den zugehörigen Restaurants Take-away-Speisen an. Ein Drittel der 1,2 Mio. Konsument*innen pro Woche nutzt laut einem von Migros betriebenen „community Blogbeitrag“ die Take-Away-Option (Migros 2019). Die Einwegbehälter, die genutzt werden, bestehen aus Polyethylenterephthalat (PET) und wiegen ca. 30 g pro Tasse (Migros 2019). Dies führt zu 624 t Kunststoffabfall pro Jahr. Seit 2017 werden auch Mehrwegschalen in einem Pfandsystem angeboten. Konsument*innen können diese bei allen teilnehmenden Migros Filialen zurückgeben. Diese wiegen ca. 235 g pro Schale und können mindestens 100 Mal genutzt werden (Migros 2019). Unter der Annahme, dass der Anteil jener 1,2 Mio. Konsument*innen, der Take-away in Anspruch nimmt, die Mehrweglösung nutzt, führt dies zu 48,9 t Kunststoffabfall pro Jahr. Somit können durch den Einsatz von Mehrwegbehältern theoretisch 92,2 % des durch ein Unternehmen verursachten Kunststoffabfalls verhindert werden.

In Tab. 3 sind diese Ergebnisse tabellarisch dargestellt. Die Ergebnisse beziehen sich auf die verursachte Abfallmenge und berücksichtigen keine weiteren Faktoren. Weitere Ergebnisse, wie zum Beispiel ein ökobilanzieller Vergleich der beiden Behältertypen, sind bis dato ausständig.

**Tab. 3:** Vergleich der Abfallmengen durch Einweg- und Mehrwegbehälter am Beispiel der Migros-Gruppe; Ergebnisse wurden gerundet

Behältertyp	Material	Gewicht in g	Nutzungen	Abfall pro Nutzung in g	Abfall in t/a
Einweg	PET	30	1	30	624
Mehrweg	PPT	235	100	2,4	48,8

Am Beispiel Migros werden das Potenzial, Abfälle durch Mehrweglösungen zu minimieren, und die damit verbundenen Herausforderungen klar erkenntlich. Die flächendeckende Einführung von Mehrwegbehältern könnte schweizweit 575,2 t Kunststoffabfall pro Jahr in der Schweiz einsparen. Das entspricht einer Reduktion um 92,2 % des Einwegverpackungsabfalls, der durch Migros mitverursacht wird. Die 20.800.000 Einwegschalen würden dabei gegen 208.000 Mehrwegschalen ausgetauscht werden. Diese würden je Behälter 100 Mal verwendet, gespült, getrocknet und gelagert werden müssen. Und dies alles unter hygienischen Bedingungen, die die anschließende Befüllung mit verschiedensten Lebensmitteln ermöglicht. Da in den meisten Ausgabestellen für Take-away der Migros-Kette auch klassischer Gaststättenbetrieb angeboten wird, dürfte dies für Migros mit marginalem Mehraufwand möglich sein. Die tatsächliche Nutzung von Mehrwegbehältern liegt, laut Telefonat mit einer Vertreterin der Migroskette, jedoch unter 10 %. Gründe für die geringe Nutzung der Mehrwegalternativen werden im Abschnitt „Herausforderungen an Mehrwegsysteme“ diskutiert. Ob ein genereller Umstieg auf Mehrwegbehälter für das Take-away-Geschäft im Sinne einer Ökobilanz tatsächlich günstig ausfällt, ist zwar wahrscheinlich, wurde jedoch noch nicht ermittelt.

reCircle, ein weiterer Mehrweganbieter aus der Schweiz, zeigt ebenfalls das Potenzial für Mehrwegbehälter im Bereich des Convenience Food auf. Seit 2016 bietet dieser, als Verein organisierte Anbieter ein pfandbasiertes Mehrwegsystem für Take-away-Speisen an. Laut Angaben auf der reCircle-Homepage werden dadurch bis zu 50.000 Einwegschalen pro Tag ersetzt. Die derzeit 1000 Partnerunternehmen zahlen dafür eine Systemgebühr und entrichten das Pfand für die benötigte Menge an Mehrwegbehältern. Konsument*innen hinterlegen 10 CHF als Pfand. Die Rückgabe für Konsument*innen ist bei allen teilnehmenden Betrieben möglich. reCircle setzt dabei auf Polybutylenterephthalat mit 30 % Glasfaser (PBT GF30). Die Wahl fiel auf dieses Material aufgrund der höheren Härte im Vergleich zu PP. Diese ist ausschlaggebend, um möglichst viele Nutzungszyklen einzelner Behälter zu gewährleisten. Darüber verweist reCircle auf eine Ökobilanz für ihre Mehrwegbehälter, die eine positive Bilanz ab 10 Waschzyklen aufweisen (Homepage recircle). Die Rückgabehäufigkeit der Behälter liegt derzeit bei ca. 20 %. Die meisten der Nutzer*innen nutzen also eine „Bowl“ öfter. Die Motivation des Vereins ergibt sich aus Litteringstudien, die seit 2004 in der Schweiz durchgeführt wurden (Heeb et al. 2004). Hier wurde erhoben, dass mit Take-away assoziiertes Littering, Take-away-Verpackungen und Getränkeverpackungen einen Anteil von 52 % am gelitterten Material ausmachen.

Rebowl ist ein Anbieter für ein pfandbasiertes Mehrwegsystem aus Deutschland und gehört zur Marke Recup. Diese bietet deutschlandweit ein pfandbasiertes Mehrwegbechersystem für Coffee-To-Go an. Ein Pilotversuch wurde von Rebowl 2019 in München und Köln gestartet. In diesen beiden Städten wird das Projekt mit einer zweiten Testphase ab Januar 2020 weitergeführt. Seit 2020 gibt es auch einen Pilotversuch in Berlin. Ziel des Pilotversuchs ist es, ein Pfandsystem zu etablieren, das möglichst breit angenommen wird. Derzeit wird als Material für die Schüsseln PP genutzt. Das Unternehmen arbeitet an einer optimierten Materiallösung, da PP sehr weich und damit nicht kratzbeständig ist. Der deutschlandweite Start erfolgte 2020.

Tiffin Loop (früher Tiffinprojekt) ist ein Berliner Projekt, das Take-away-Schüsseln aus Stahl anbietet. 2015 wurde es durch Crowdfunding ermöglicht. Das Projekt übernahm ein Prinzip, das in Indien bereits seit Jahrzehnten bekannt ist. Take-away-Essen wird dort in wiederverwendbaren, praktisch unzerstörbaren Stahlbehältern ausgegeben.

### Herausforderungen an Mehrwegsysteme

Für Anbieter, die vorgekochte Speisen anbieten und verstärkt auf Take-away setzen, ist der logistische Mehraufwand des Spülens, Trocknens, Lagerns etc. eine ernstzunehmende Herausforderung. Nicht in allen kleinen Restaurants oder Filialen einer LEH-Kette gibt es die Möglichkeit, vor Ort eine große Menge an Mehrwegbehältern zu spülen bzw. die gespülten Behälter zu trocknen und zu lagern. Auch das Annehmen von Behältern und die Ausgabe des Pfands sowie die anschließende Lagerung der noch zu reinigenden Behälter verursachen zusätzlichen Arbeitsaufwand.

Weitere Beispiele sind Gastronomiebetriebe, deren Funktionsprinzip auf Einweg und Take-away basiert, wie zum Beispiel klassische Fast-Food-„Restaurants“. Auch auf Auslieferung spezialisierte Betriebe werden dadurch vor Herausforderungen gestellt. Einerseits kann als Beispiel der oben genannte Pizzalieferservice genannt werden und andererseits sogenannte „Ghostkitchens“. Letztere bieten exklusiv Lieferservices an und verzichten aus Gewinnmaximierungsgründen komplett auf Gaststättenbetrieb (Sherred 2019). Die meisten kooperieren mit Third-Party-Delivery-Unternehmen oder werden von diesen aufgekauft (Ksienrzyk 2019).

Für solche Anbieter müssen zentralisierte Spül- und Logistikservices diese Aufgaben als Dienstleistung übernehmen. Bereits vorhandene Infrastruktur und logistische Expertise sind hierbei von Vorteil. Dies zeigt auf, dass durch kreislaufwirtschaftliche Prinzipien wie jenes der Wiederverwendung auch Arbeitsplätze generiert werden können (Altendorfer et al. 2019). Solche Spülzentren sollten möglichst nah an Ballungsräumen liegen, da dort mit dem größten Bedarf zu rechnen ist. Außerdem werden die Wege und damit die CO_2_-Emissionen verringert, was durchaus im Sinne einer nachhaltigen Lösung sein sollte.

Im Take-away-Bereich gibt es trotz diverser Herausforderungen bereits mehrere Anbieter von Mehrweglösungen, die neben den Behältertypen auch funktionierende Systeme anbieten, um Einwegverpackungsabfälle zu reduzieren. Dennoch liegt die tatsächliche Nutzung von Mehrwegbehältern im niedrigen einstelligen Prozentbereich gegenüber den Einweglösungen. Sowohl im Rahmen persönlicher Kommunikation mit Vertreter*innen sowohl der Migroskette als auch von reCircle sowie in bestehender Literatur zur artverwandten Fragestellung der Einweg- vs. Mehrwegbecher (Kauertz et al. 2019) wird als wichtigster Grund hierfür das Vorhandensein zweier Behältertypen vermutet. Solange Einweg- und Mehrwegbehälter gleichzeitig angeboten werden, wird der Wechsel hin zu Mehrweg gehindert; dies wird auch für Coffee-To-Go-Becher vermutet (Kauertz et al. 2019). Der Gesetzgeber könnte die Nutzung von Einwegbehältern verbieten, was jedoch massive Einschnitte in verschiedene Wirtschaftsströme bedeuten würde und damit mit immensem Widerstand behaftet wäre. Dies ergibt sich aus einer Befragung, die im Rahmen der o. g. Studie des deutschen Umweltbundesamtes (Kauertz et al. 2019) durchgeführt wurde. In diesem Kontext können Unternehmen also den proaktiven Schritt setzen und nur mehr die Mehrweglösung anbieten. Dies ist zwar ein gewagter Schritt, wird aber im Coffee-To-Go-Bereich bereits von einem kleinen Tiroler Betrieb umgesetzt (Korkmaz 2020). Auch wenn dies noch keine flächendeckende Einführung bedeutet, kann ein solcher Alleingang durchaus als Leuchtturmprojekt betrachtet werden.

Eine weitere Option, um die Umstellung zu beschleunigen, könnten Anreizsysteme sein. So kann die Nutzung eines Einwegbehälters mit zusätzlichen Kosten verknüpft und damit das Produkt teurer gemacht werden (negative Incentives). Die Mehreinnahmen können dann genutzt werden, um die höheren Sammel‑, Entsorgungs- und Behandlungskosten durch potenzielles Littering und das verstärkte Abfallaufkommen zu kompensieren. Negative Anreizsysteme funktionieren allerdings nicht so gut wie positive. In diesem Sinne könnte angedacht werden, das Produkt im Falle der Pfandbehälternutzung zu verbilligen. Beispiele aus dem Coffee-To-Go-Bereich zeigen, dass dies in einer wahrnehmbaren Mehrnutzung der Mehrwegalternativen mündet (Kauertz et al. 2019; Knapp and Bockreis 2019).

Um eine generelle Beteiligung überhaupt erst zu ermöglichen, darf der Pfandpreis nicht zu hoch angesetzt werden, um Pfandschlupf zu verhindern, aber auch nicht zu niedrig.

Letztlich können bewusstseinsbildende Maßnahmen dazu beitragen, einerseits Konsument*innen und andererseits auch das Verkaufspersonal, für die Nutzung von Mehrwegalternativen zu sensibilisieren. Das Verkaufspersonal ist jener Teil eines Unternehmens, das den größten Kundenkontakt aufweist. Deshalb sind es eben jene Angestellten, die durch entsprechende Schulungen am besten geeignet sind, die Mehrwegalternativen an die Kund*in zu bringen (Kauertz et al. 2019). Dieses Vorgehen wird jedoch nur zielführend sein, wenn die gesamte Unternehmensstruktur entsprechend eingebunden wird.

Ein weiteres Hindernis stellt die Rückgabemotivation der Konsument*innen dar. Bei den betrachteten Systemanbietern liegt diese nur bei 10–20 %; diese Quoten wurden von Vertreter*innen der Migroskette und reCircle während persönlicher Kommunikation geschätzt. Im ökologischen Sinne könnte dies auf den ersten Blick akzeptabel erscheinen, unter der Annahme, dass die Konsument*innen die Mehrwegbehälter wohl auch privat als Ersatz für Einwegverpackungen nutzen. Für die Wirtschaftlichkeit eines pfandbasierten Mehrwegsystems ist dies jedoch nicht zielführend. Die nicht zurückgegebenen Behälter müssen durch Neukauf kompensiert werden, was wiederum eine übermäßige Produktion bedingt. Dies führt zu erhöhtem Ressourcenverbrauch und egalisiert damit zumindest zum Teil den positiven Effekt der Mehrwegbehälter. Eine mögliche Lösung wäre es, vermehrt Bewusstsein für Mehrwegsysteme zu schaffen. Dies wird zum Beispiel durch entsprechende Kampagnen ermöglicht, die im Vorfeld einer Einführung ablaufen. Die Kampagnen müssen ein möglichst breites Zielpublikum in Bezug auf Alter, Bildungstand, Einkommen etc. ansprechen.

Zu diesen Herausforderungen kommt außerdem, dass pfandbasierte Mehrwegsysteme im Convenience-Food-Bereich erst seit 2015 aktiv und damit noch in der Anfangsphase sind. Systemimmanente Schwierigkeiten haben deshalb immer wieder zum Verschwinden von Mehrweganbietern geführt. Ein gescheiterter Betrieb beschrieb in einem Telefonat, was nicht funktionierte und zum Scheitern führte:

Wir setzten auf eine Leihgebühr von 0,5 € pro Tag, anstatt ein Pfandsystem zu nutzen. Die Leihgebühr hat sich als nicht zielführend erwiesen, da Konsument*innen den Preis dafür nicht zahlen wollten. Darüber hinaus erwiesen sich die nicht vorhandene App bzw. das generelle Fehlen webbasierter Angebote als weiterer Hinderungsgrund. Letztlich war es das Karteikartensystem, das zu langen Wartezeiten bei der Aus- und Rückgabe der Mehrwegbehälter führte und somit nicht in ein convenience-basiertes Angebot passten, welches zur Schließung des Unternehmens führte.

Insbesondere für Unternehmen, die auf pfandbasierte Mehrwegsysteme umsteigen möchten, ergeben sich zusätzliche Herausforderungen in Bezug auf Lagerlogistik, Transportlogistik, Reinigung, Pfandhöhe und Umverteilung der Pfandrückzahlungen. Diese sollten im Vorfeld einer flächendeckenden Einführung erhoben und im Rahmen überschaubarer Pilotphasen getestet und optimiert werden.

Gegenwärtig ist es schwer abzuschätzen, in welchen Bereichen Mehrwegbehälter die Einwegalternativen tatsächlich ersetzen werden können.

So ist, laut Telefonaten mit einer Vertreterin der Firma Gigatherm, die Mehrwegnutzung folierter Behälter, wie sie für vorgekochte, aber noch zu erwärmende Speisen üblich ist, nach derzeitigem Stand der Technik nicht möglich. Dies ist der thermischen Versiegelungstechnik geschuldet, die auf einer chemischen Bindung zwischen Behälter und Folie basiert. Diese Versiegelung ist notwendig, um das Mindesthaltbarkeitsdatum und hygienische Standards zu garantieren. Durch das Wärmen der Speisen, Waschvorgänge und die genutzten Lösungsmittel wird die für diese Versiegelung notwendige Klebeschicht auf Seiten des Behälters zerstört. Diese Tatsache ist insbesondere für die fertigende Industrie interessant, da es offensichtlich Bedarf an entsprechenden Lösungen gibt. Zwar gibt es Möglichkeiten, durch Einsatz von Ventilen auch Mehrwegbehälter aus Glas entsprechend zu versiegeln, diese sind jedoch teuer und bei weitem nicht so stabil wie die diversen Kunststofflösungen. Ein weiteres Hindernis in diesem Zusammenhang ist, dass die meisten Unternehmen bereits über technisch aktuelle Einwegverpackungslösungen verfügen und diese aus finanziellen und oder logistischen Gründen nicht austauschen können oder wollen. Dies wurde von mehreren Branchenvertreter*innen bestätigt.

Ein weiteres Kriterium betrifft die Festigkeit der Behälter. Die Materialien müssen so gewählt werden, dass sie möglichst langlebig, lebensmittelkompatibel und leicht sind. Im leeren Zustand müssen sie stapelbar sein, um Platz zu sparen. Sie müssen leicht zu reinigen und zu trocknen sein. Diese Ansprüche werden vor allem durch diverse Kunststoffe erfüllt, auch wenn nicht alle Kunststoffe dafür geeignet sind. So hat sich in diversen Pilotphasen einiger Unternehmen gezeigt, dass PP aufgrund zu hoher Kratzanfälligkeit eigentlich nicht als Material für Mehrwegbehälter geeignet ist.

In Bezug auf Kunststoffe und Lebensmittel werden darüber hinaus immer häufiger Bedenken geäußert. Diese betreffen vor allem die Migration, also das Wandern, chemischer Additive aus den Verpackungen in die Lebensmittel (Ernstoff et al. 2018, 2017; Guerreiro et al. 2018).

Aber auch die mögliche Kontamination mit Mikrokunststoffen (Iniguez et al. 2017; Ossmann et al. 2018) wirft die Frage auf, ob und – wenn ja – welche Kunststoffe künftig für den Einsatzbereich im Lebensmittelmarkt geeignet sind.

Insbesondere unter der Annahme der Wiederverwendung von Kunststoffbehältern für die o. g. Wirtschaftszweige gilt es die bisher nicht hinreichend untersuchte mögliche Ablösung von Mikro- und Nanopartikeln zu berücksichtigen. Diese erhöht sich durch die wiederholte Nutzung und die regelmäßigen Spülzyklen. Derzeit sind keine Mikroplastik-assoziierten Informationen aus Ökobilanzsoftware bekannt. Damit fehlt ein wichtiges Bilanzierungstool für künftige Kunststoffproduktanalysen.

Selbst bei Kartonagen gibt es Bedenken bezüglich möglicher Migration chemischer Schadstoffe aus der Verpackung in die Lebensmittel (Bengtstrom et al. 2016; Nguyen et al. 2017; Yuan et al. 2016).

Über die negativen Aspekte von Aluminium wurde ebenfalls bereits viel berichtet (Stahl et al. 2017, 2018), und es scheint daher ebenfalls ungeeignet für eine flächendeckende Anwendung.

Lediglich bezüglich Stahlbehältern im Lebensmittelberreich konnte keine Publikation über negative Auswirkungen gefunden werden. Damit scheint Stahl eine geeignete Zukunftsoption darzustellen.

## Schlussfolgerungen

Der Convenience-Food-Bereich, inklusive Take-away und Lieferdiensten, wird weiterwachsen. Dabei werden zukünftig neben den klassischen und bekannten Mitnehmoptionen aus Lebensmitteleinzelhandel und Gastronomie zwei Geschäftsmodelle relevant sein.

1. Anbieter, die über keine eigene Gaststube mehr verfügen. Bestes Beispiel hierfür ist der Kauf eines entsprechenden Startups durch Delivery Hero (Ksienrzyk 2019). Diese sogenannten „Ghostkitchens“ können aufgrund des fehlenden „Overheads“ (alle mit Gaststättenbetrieb assoziierten, nicht steuerlich absetzbaren Kosten) hohe Gewinne erzielen (Glorioso et al. 2015; Sherred 2019).
2. Third-Party-Delivery-Unternehmen. Diese verfügen über ausgeklügelte Logistik und die notwendige Erfahrung, Essenslieferungen als Dienstleistung anzubieten. Darüber hinaus wären diese Unternehmen aufgrund dieser logistischen Vorteile auch prädestiniert, entsprechende pfandbasierte Mehrweglösungen anzubieten. Die Kosteneffizienz wird neben den logistischen Herausforderungen die Hauptantriebskraft hinter der Einführung von Mehrwegsystemen sein.

Diese Wirtschaftszweige nachhaltig, klimapositiv und abfallvermeidend weiter zu entwickeln wird unter anderem durch pfandbasierte Mehrwegbehälter möglich sein. Diese Behälter müssen jedoch aus einem Material bestehen, das neben den Anforderungen der Wirtschaft auch jenen der Umwelt gerecht wird. Um dies zu gewährleisten, ist noch Forschungsbedarf im Bereich der Wiederverwendung diverser Materialien gegeben.

## References

[CR41] **Abfallaufkommen. Hg. v. Umweltbundesamt. Umweltbundesamt (2019):** https://www.umweltbundesamt.de/daten/ressourcen-abfall/abfallaufkommen#deutschlands-abfall. Zugegriffen am: 03.09.2020

[CR1] **Altendorfer M., Pomberger R., Gelbmann U. (2019):** Vergleich abfallwirtschaftlicher Systeme für Siedlungsabfälle mit Schwerpunkt Beschäftigungseffekte Österreichische Wasser- und Abfallwirtschaft 72:21–37. 10.1007/s00506-019-00627-x

[CR2] **Bengtstrom L. et al. (2016):** Non-targeted screening for contaminants in paper and board food-contact materials using effect-directed analysis and accurate mass spectrometry Food Addit Contam Part A Chem Anal Control Expo Risk Assess 33:1080–1093. 10.1080/19440049.2016.1184941 27146477 10.1080/19440049.2016.1184941

[CR3] **Chalupova L. (2019):** Beitrag von Betriebsrestaurants zum nachhaltigen Konsum am Beispiel vom Abfall. In: Aktuelle Ansätze zur Umsetzung der UN-Nachhaltigkeitsziele. pp 313–334. 10.1007/978-3-662-58717-1_17

[CR4] **Christoforou N. (2019):** P8_TA-PROV(2019)0305 Reduction of the impact of certain plastic products on the environment ***I.

[CR5] **Delivery Hero AG (2017):** Delivery Hero Geschäftsbericht 2017.

[CR6] **DUH (2020):** Deutsche Umwelthilfe beantragt in 64 Städten ein Ende der Einwegmüll-Flut. Deutsche Umwelthilfe, Online

[CR7] **EC (2015):** A European Strategy for Plastics in a Circular Economy.

[CR8] **Enzinger S. (2019):** Chance Kreislaufwirtschaft. Umweltbundesamt, Wien

[CR9] **Ernstoff A., Niero M., Muncke J., Trier X., Rosenbaum R.K., Hauschild M., Fantke P. (2018):** Challenges of including human exposure to chemicals in food packaging as a new exposure pathway in life cycle impact assessment The International Journal of Life Cycle Assessment 24:543–552. 10.1007/s11367-018-1569-y

[CR10] **Ernstoff A.S., Fantke P., Huang L., Jolliet O. (2017):** High-throughput migration modelling for estimating exposure to chemicals in food packaging in screening and prioritization tools Food Chem Toxicol 109:428–438. 10.1016/j.fct.2017.09.024 28939300 10.1016/j.fct.2017.09.024

[CR42] **Van Eygen E., Fellner J. (2019):** Nutzen und Kosten eines verstärkten Recyclings von Kunststoffverpackungen Österreichische Wasser- und Abfallwirtschaft 72:38-46. 10.1007/s00506-019-00629-9

[CR11] **Fischer T. (2020):** „Einwegverpackungen sind durch die Decke geschossen“. Deutschlandfunk,

[CR12] **Gallego-Schmid A., Mendoza J.M.F., Azapagic A. (2019):** Environmental impacts of takeaway food containers Journal of Cleaner Production 211:417–427. 10.1016/j.jclepro.2018.11.220

[CR13] **Geyer R., Jambeck J.R., Law K.L. (2017):** Production, use, and fate of all plastics ever made SCIENCE ADVANCES 3. 10.1126/sciadv.1700782 10.1126/sciadv.1700782 PMC5517107 28776036

[CR14] **Gill M.B. et al. (2020):** Consumer preferences for eco-friendly attributes in disposable dinnerware Resources, Conservation and Recycling 161. 10.1016/j.resconrec.2020.104965

[CR15] **Glorioso C., Givens A., Stulberger E. (2015):** I‑Team: Restaurants Use False Identities on Food Delivery Websites.

[CR16] **Grundberg C. et al. (2018):** Is The Kitchen Dead? UBS Evidence Lab, UBS South Africa

[CR17] **Guerreiro T.M., de Oliveira D.N., Melo C., de Oliveira Lima E., Catharino R.R. (2018):** Migration from plastic packaging into meat Food Res Int 109:320–324. 10.1016/j.foodres.2018.04.026 29803455 10.1016/j.foodres.2018.04.026

[CR18] **Hahladakis J.N., Iacovidou E. (2018):** Closing the loop on plastic packaging materials: What is quality and how does it affect their circularity? Sci Total Environ 630:1394–1400. 10.1016/j.scitotenv.2018.02.330 29554759 10.1016/j.scitotenv.2018.02.330

[CR44] **Heeb, Johannes; Hoffelner, Wolfgang; Christen, Marius; Hadinia, Kurosch; Labhardt, Sarah; Wunderli, Rahel et al. (2004):** litteringstudie-1-2004. Hg. v. BUWAL, Stadt Basel, Bern, Illnau-Effretikon, Lausanne und Zürich, Schweizerischer Städteverband.

[CR19] **Heubacher A. (2018):** 24.000 Tonnen: Tirols Plastikmüllberg wächst. Innsbruck

[CR20] **Hietler P., Pladerer C. (2020):** Littering in Österreich als Beitrag für Mikro- und Makrokunststoffe in der Umwelt Österreichische Wasser- und Abfallwirtschaft 72:370–377. 10.1007/s00506-020-00699-0

[CR21] **Iniguez M.E., Conesa J.A., Fullana A. (2017):** Microplastics in Spanish Table Salt Sci Rep 7:8620. 10.1038/s41598-017-09128-x 28819264 10.1038/s41598-017-09128-x PMC5561224

[CR22] **Istel K., Detloff K. (2018):** Einweggeschirr und To-Go-Verpackungen Abfallaufkommen in Deutschland 1994 bis 2017.

[CR23] **Kauertz B., Schlecht S., Markwardt S., Knappe F., Reischl S., Pauer G. (2019):** Untersuchung der ökologischen Bedeutung von Einweggetränkebechern im Außer-Haus-Verzehr und mögliche Maßnahmen zur Verringerung des Verbrauchs. Umweltbundesamt,

[CR24] **Kedzierski M. et al. (2018):** Threat of plastic ageing in marine environment. Adsorption/desorption of micropollutants Mar Pollut Bull 127:684-694. 10.1016/j.marpolbul.2017.12.059 29475712 10.1016/j.marpolbul.2017.12.059

[CR25] **Knapp J., Bockreis A. (2019):** MehrWert für Innsbruck - Coffee-to-go im MehrWegbecher. Universität Innsbruck,

[CR26] **Korkmaz C. (2020):** Homepage Coffekult.

[CR27] **Ksienrzyk L. (2019):** Delivery Hero kauft Berliner Ghost-Restaurant-Startup.

[CR28] **Lechleitner A., Schwabl D., Schubert T., Bauer M., Lehner M. (2019):** Chemisches Recycling von gemischten Kunststoffabfällen als ergänzender Recyclingpfad zur Erhöhung der Recyclingquote Österreichische Wasser- und Abfallwirtschaft 72:47-60. 10.1007/s00506-019-00628-w

[CR29] **Migros J. (2019):** Weg mit dem Wegwerfgeschirr - Migros Community.

[CR30] **Nguyen P.M., Julien J.M., Breysse C., Lyathaud C., Thebault J., Vitrac O. (2017):** Project SafeFoodPack Design: case study on indirect migration from paper and boards Food Addit Contam Part A Chem Anal Control Expo Risk Assess 34:1703-1720. 10.1080/19440049.2017.1315777 28374636 10.1080/19440049.2017.1315777

[CR31] **Ossmann B.E., Sarau G., Holtmannspotter H., Pischetsrieder M., Christiansen S.H., Dicke W. (2018):** Small-sized microplastics and pigmented particles in bottled mineral water Water Res 141:307-316. 10.1016/j.watres.2018.05.027 29803096 10.1016/j.watres.2018.05.027

[CR32] **Schinkel J., Wilts H. (2020):** Kunststoffabfälle von Verpackungen und Einwegprodukten durch Multi-Akteurs-Partnerschaften verringern Müll und Abfall 8:6

[CR33] **Schüler K. (2018):** GVM-Studie-Einweggeschirr-Sofortverzehr. Gesellschaft für Verpackungsmarktforschung,

[CR34] **Sherred K. (2019):** Why ‘ghost’ restaurants are changing the delivery game. industryDive,

[CR35] **Single H. (2019):** Pizzabow.

[CR36] **Sokola I. (2020):** Höheres Plastikmüllaufkommen in Corona-Krise. Die Zeit, Online

[CR37] **Song G., Zhang H., Duan H., Xu M. (2018):** Packaging waste from food delivery in China’s mega cities Resources, Conservation and Recycling 130:226-227. 10.1016/j.resconrec.2017.12.007

[CR38] **Stahl T. et al. (2017):** Migration of aluminum from food contact materials to food‑a health risk for consumers? Part III of III: migration of aluminum to food from camping dishes and utensils made of aluminum Environ Sci Eur 29:17. 10.1186/s12302-017-0117-x 28458987 10.1186/s12302-017-0117-x PMC5388722

[CR39] **Stahl T., Falk S., Taschan H., Boschek B., Brunn H. (2018):** Evaluation of human exposure to aluminum from food and food contact materials European Food Research and Technology 244:2077-2084. 10.1007/s00217-018-3124-2

[CR40] **Technisches Büro Hauer (2020):** Analysen des Restabfalls in Tirol 2018/19. Tiroler Landesregierung, Innsbruck, Korneuburg

[CR43] **Yuan G., Peng H., Huang C., Hu J. (2016):** Ubiquitous Occurrence of Fluorotelomer Alcohols in Eco-Friendly Paper-Made Food-Contact Materials and Their Implication for Human Exposure Environ Sci Technol 50:942-950. 10.1021/acs.est.5b03806 26655429 10.1021/acs.est.5b03806

